# Effect of Low and High Doses of Two Selective Serotonin Reuptake Inhibitors on Pregnancy Outcomes and Neonatal Mortality

**DOI:** 10.3390/toxics10010011

**Published:** 2022-01-01

**Authors:** Rafael R. Domingues, Hannah P. Fricke, Celeste M. Sheftel, Autumn M. Bell, Luma C. Sartori, Robbie S. J. Manuel, Chandler J. Krajco, Milo C. Wiltbank, Laura L. Hernandez

**Affiliations:** 1 Department of Animal and Dairy Sciences, University of Wisconsin-Madison, Madison, WI 53706, USA; reisdomingue@wisc.edu (R.R.D.); hfricke@wisc.edu (H.P.F.); underriner@wisc.edu (C.M.S.); ambell3@wisc.edu (A.M.B.); canavessisar@wisc.edu (L.C.S.); rmanuel@wisc.edu (R.S.J.M.); ckrajco@wisc.edu (C.J.K.); wiltbank@wisc.edu (M.C.W.); 2 Endocrinology and Reproductive Physiology Program, University of Wisconsin-Madison, Madison, WI 53706, USA; 3 Molecular and Cellular Pharmacology Program, University of Wisconsin-Madison, Madison, WI 53706, USA

**Keywords:** selective serotonin reuptake inhibitor, perinatal mortality, neonatal morbidity, fluoxetine, sertraline

## Abstract

Selective serotonin reuptake inhibitors (SSRI) are the most common antidepressant used by pregnant women; however, they have been associated with adverse pregnancy outcomes and perinatal morbidity in pregnant women and animal models. We investigated the effects of two SSRI, fluoxetine and sertraline, on pregnancy and neonatal outcomes in mice. Wild-type mice were treated daily with low and high doses of fluoxetine (2 and 20 mg/kg) and sertraline (10 and 20 mg/kg) from the day of detection of a vaginal plug until the end of lactation (21 days postpartum). Pregnancy rate was decreased only in the high dose of fluoxetine group. Maternal weight gain was reduced in the groups receiving the high dose of each drug. Number of pups born was decreased in the high dose of fluoxetine and low and high doses of sertraline while the number of pups weaned was decreased in all SSRI-treated groups corresponding to increased neonatal mortality in all SSRI-treated groups. In conclusion, there was a dose-dependent effect of SSRI on pregnancy and neonatal outcomes in a non-depressed mouse model. However, the distinct placental transfer of each drug suggests that the effects of SSRI on pup mortality may be mediated by SSRI-induced placental insufficiency rather than a direct toxic effect on neonatal development and mortality.

## 1. Introduction

Psychotropic medications that are taken during pregnancy can pose risks of toxic effects to both mother and fetus [1,2]. About 8–12% of pregnant women take antidepressants [2,3,4] and selective serotonin reuptake inhibitors (SSRI) are the most commonly used antidepressant [4,5]. Among SSRI, fluoxetine was the first clinically available and remains one of the most popular while sertraline is currently the most prescribed SSRI to pregnant women [6]. Although teratogenic effects of some SSRI (i.e., paroxetine) are well recognized [7], other SSRI (sertraline, citalopram, fluoxetine) continue to be commonly prescribed to pregnant women [6]. Nevertheless, in the past decades multiple studies highlighted the association between SSRI use during gestation and adverse maternal, fetal, and neonatal health outcomes including decreased birthweight, preterm birth, and increased perinatal morbidity and mortality [3,6,8,9].

In addition to its role as a neurotransmitter, serotonin is a hormone with vasoactive properties [10] so that increased serotonin signaling selectively increases vascular resistance in the uterus causing reduced uterine vascular perfusion [11]. SSRI increase free (plasma) serotonin content by inhibiting serotonin uptake into platelets [12], thereby also decreasing uterine vascular perfusion [13]. Reduced uteroplacental blood flow leads to placental dysfunction/insufficiency, the main cause of fetal growth restriction [14,15]. The role of serotonin and SSRI on fetal growth restriction have been reviewed [10,15,16,17,18]. Fetal growth restriction is an important cause of prematurity, perinatal morbidity, and lifelong health impairment in addition to being the second leading cause of perinatal mortality [19,20].

Although fluoxetine and sertraline inhibit the serotonin transporter (SERT), their pharmacokinetics differ quite markedly [21]. Following oral ingestion, fluoxetine exhibits greater bioavailability compared to sertraline (80% vs. 44%, respectively) [21]. Plasma concentrations of sertraline follow linear kinetics (increased dose promotes proportional increase in systemic concentrations of the drug) [22]. However, fluoxetine follows nonlinear kinetics resulting in a disproportional increase in systemic concentrations after dose augmentation. Additionally, while sertraline has a half-life of 22 to 36 h, fluoxetine has a half-life of 1–6 days [22]. Furthermore, fluoxetine metabolism produces an active metabolite, norfluoxetine, which has a longer half-life than fluoxetine itself (8–15 days) while sertraline’s metabolites are essentially inactive. Lastly, placental transfer of fluoxetine is greater compared to sertraline (70% vs. 25%) [23,24]. It is unclear whether these two popular SSRI with distinct kinetics similarly affect pregnancy outcomes and neonatal morbidity/mortality, particularly given their distinct placental transfer.

Because of the widespread use of SSRI during gestation and their possible detrimental effects on pregnancy and neonatal outcomes, we aimed to compare the effects of low and high doses of fluoxetine and sertraline on pregnancy and neonatal outcomes. Pregnant mice were treated with SSRI during the second half to pregnancy. Altogether, both low and high doses of sertraline and fluoxetine adversely affected neonatal outcomes; however, only the high dose of fluoxetine resulted in decreased pregnancy rate.

## 2. Materials and Methods

### 2.1. Animals

All experiments were approved by the Research Animal Care and Use Committee at the University of Wisconsin-Madison and were performed under protocol number A005789-A01. Mice were housed in a controlled environmental facility for biological research in the Biochemistry Department vivarium (fluoxetine study) and the Animal and Dairy Sciences Department vivarium (sertraline study) at the University of Wisconsin-Madison. Animal facilities were maintained at a temperature of 25 °C and a humidity of 50% to 60%, with a 12:12 h light-dark cycle with ad libitum water and food (LabDiet 5015, TestDiet, Richmond, IN, USA). Wild-type C57BL/6J mice were obtained from Jackson Laboratories (stock # 000664, Jackson Laboratories, Bar Harbor, ME, USA). Females included in our study either originated from Jackson Laboratories or were F1 offspring from our breeding colony. Beginning at 6 weeks of age, female mice were bred with a male overnight.

### 2.2. Experimental Design

After detection of a vaginal plug (day post coitum [DPC] 0.5), dams were individually housed and randomly assigned to treatment groups. All mice received a daily intraperitoneal injection between the hours of 0800 and 0900 of either vehicle, low and high dose of SSRI (either fluoxetine or sertraline) from DPC 0.5 until the end of lactation (21 days postpartum). Virgin females (unmated; 2–6 per group) were treated with vehicle, low and high dose of SSRI (either of fluoxetine or sertraline) for evaluation of the effect of SSRI on weight in nonpregnant mice. Virgin mice received seven treatments, equivalent to pregnant dams treated from DPC0.5 to 6.5. All mice were weighed daily at time of injection. A successful pregnancy was determined by weight gain between DPC 0.5 and 7.5 [25] and confirmed by parturition. Pregnancy length and number of pups born were recorded on the day of parturition. The number of live pups was recorded daily during lactation. Litters were not standardized.

For the fluoxetine study, fluoxetine hydrochloride (F312; Sigma-Aldrich, St. Louis, MO, USA) was reconstituted in saline. Mice were treated with vehicle (saline; n = 28), low dose of fluoxetine (2 mg/kg; n = 32), or high dose of fluoxetine (20 mg/kg; n = 127).

For the sertraline study, sertraline hydrochloride (S6319; Sigma-Aldrich, St. Louis, MO, USA) was reconstituted in 8.3% dimethyl sulfoxide (DMSO) diluted in saline for the low dose group (10 mg/kg; n = 32) and in 15% DMSO diluted in saline for the high dose group (20 mg/kg; n = 16). To account for the different concentrations of DMSO, we had two vehicle groups: 8.3% DMSO diluted in saline (vehicle group for the low dose sertraline; n = 32) and in 15% DMSO diluted in saline (vehicle group for the high dose sertraline; n = 11).

The high dose of fluoxetine (20 mg/kg) has been extensively used in rodent studies [26,27], including reports from our laboratory [28]. However, the systemic concentrations of the drug in mice from previous studies from our laboratory were higher than in humans. Therefore, we selected a low dose (2 mg/kg) that we anticipated would be within expected systemic concentrations of fluoxetine in humans. The low dose of sertraline (10 mg/kg) was based on other reports and is expected to be within normal range of human systemic concentrations [29]. However, we had issues with drug solubility to develop a model with the higher dose of sertraline. Because we did not want to dramatically increase DMSO concentrations nor alter injection volume between studies, we were only able to treat mice at 20 mg/kg.

### 2.3. Blood Collection and Fluoxetine Assay

Blood samples were collected from the dams on DPC 0.75 and DPC 17.75. Mice were fasted for 6–8 h after the morning treatment until blood collection. Blood was collected from the submandibular vein using a 5.5 mm lancet, placed on ice for 20 min, and centrifuged at 846 g for 20 min at 4 °C; serum was stored at −80 °C until assayed. Serum fluoxetine and norfluoxetine concentrations were measured with a forensic fluoxetine ELISA kit (catalog no. 107619; Neogen, Lexington, KY, USA) according to manufacturer’s instructions, samples were diluted 1:100. A displacement curve was prepared from fluoxetine hydrochloride (S6319; Sigma-Aldrich, St. Louis, MO, USA) to create a standard curve for quantification. The cross-reactivity is 100% for fluoxetine and 67% for norfluoxetine; therefore, data is presented as fluoxetine plus norfluoxetine concentrations.

### 2.4. Statistical Analysis

All statistical analyses were performed on SAS (version 9.4; SAS Institute Inc., Cary, NC, USA). Data were analyzed with PROC MIXED procedure using one-way ANOVA and two-way ANOVA for repeated measures. Tukey HSH was used for post hoc comparisons. Studentized residuals with deviations from assumptions of normality and/or homogeneity of variance were transformed into square root, logarithms, or ranks. Survival analysis was done with PROC LIFETEST using Wilcoxon test. For the fluoxetine study, comparisons were performed among all groups. For the sertraline studies, comparisons between vehicle and treated group were performed separately for the low and high dose. A probability of ≤0.05 indicated a difference was significant and a probability between >0.05 and ≤0.1 indicated significance was approached. Data are presented as the mean ± standard error of mean (SEM).

## 3. Results

### 3.1. Systemic Fluoxetine Concentrations

Fluoxetine was undetected in vehicle-treated mice (Table 1). In fluoxetine-treated mice, there was a dose-dependent effect of treatment on systemic concentrations of fluoxetine. At 6 h after the first treatment, fluoxetine concentrations were 24-fold greater in mice treated with the high than low dose. At 6 h after the 18th treatment, fluoxetine concentrations had increased 2.4-fold with the low dose and 4.2-fold with the high dose, producing a 43-fold greater fluoxetine in the high than low dose animals.

### 3.2. Maternal Weight

**Figure 1:**
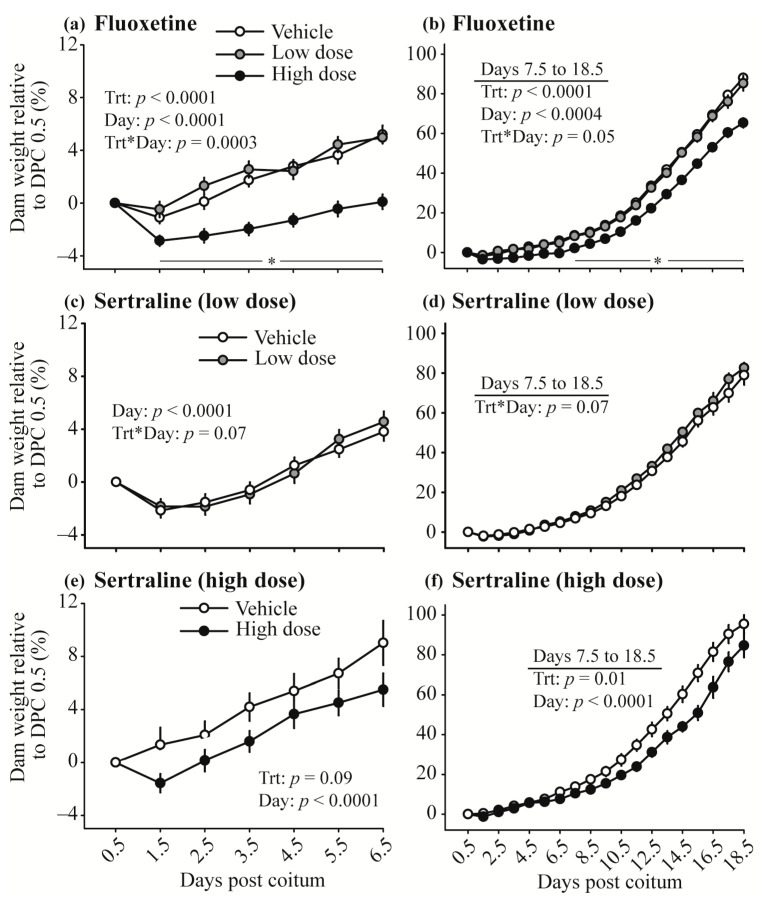
Effect of fluoxetine (**a**,**b**) and sertraline (**c**–**f**) on maternal weight gain between days post coitum (DPC) 0.5 to 6.5 (**a**,**c**,**e**) using data from pregnant (before the effect of fetal weight on maternal weight), nonpregnant, and virgin mice. Maternal weight gain (**b**,**d**,**f**) during entire gestation (DPC 0.5 to 18.5) used data from only pregnant mice with data analyzed for DPC 7.5 to 18.5. * Indicates significantly decreased weight in the high dose of fluoxetine group.

Weight gain after the onset of treatment was evaluated between DPC 0.5 to 6.5 and DPC 7.5 to 18.5. Until DPC 6.5 there is little to no effect of embryonic weight on total maternal weight [25]; therefore, pregnant, nonpregnant, and virgin mice were included in the analysis for more robust analysis of the effect of SSRI on mouse weight. In the fluoxetine study, overall weight gain during DPC 0.5 to 6.5 was decreased in the high dose group (Figure 1). Although all groups in the fluoxetine study lost weight after the first day of treatment, the high dose of fluoxetine caused greater weight loss after a single treatment compared to vehicle and low dose. Additionally, the group receiving the high dose of fluoxetine did not recuperate weight to pretreatment levels until DPC 6.5 while the vehicle and low dose groups reached pretreatment weight on DPC 2.5. Maternal weight gain was overall reduced in the high dose of fluoxetine from DPC 7.5 to 18.5. Maternal weight on the day before parturition was greatest (*p* = 0.005) in the vehicle group (32.7 ± 0.5 g), intermediate in the low dose (32.1 ± 0.7 g), and lowest in the high dose group (30.5 ± 0.4 g).

In the sertraline study, the high dose group had greater weight loss after onset of treatment and overall weight gain between DPC 0.5 and 6.5 and between DPC 7.5 and 18.5 was lower in the high dose group than vehicle. However, maternal weight on the day before parturition was not different between groups (*p* = 0.2, 32.8 ± 0.8 vs. 31.6 ± 0.7 g for the low dose; *p* = 0.6, 34.2 ± 0.5 vs. 33.5 ± 1.1 g for the high dose).

### 3.3. Pregnancy Establishment and Maintenance

In the fluoxetine study, the high dose significantly reduced pregnancy establishment (pregnancy per plug; Table 2). Sertraline treatment (low and high doses) had no significant effect on the number of pregnant mice. Gestation length was not affected by fluoxetine treatment, but sertraline extended the mean gestation length.

### 3.4. Neonatal Outcomes and Pup Survival

**Figure 2:**
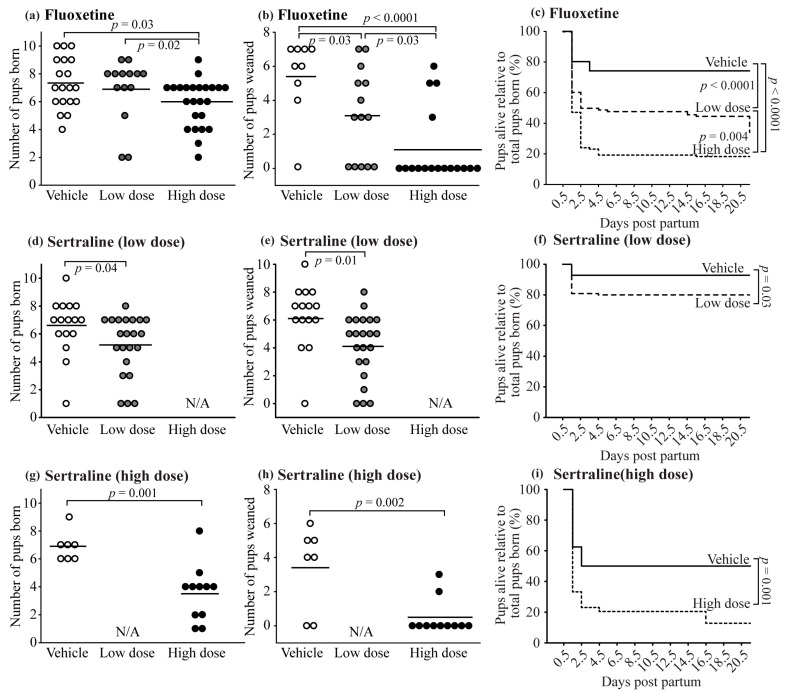
Effect of fluoxetine (**a**–**c**) and sertraline (**d**–**i**) on neonatal outcomes: Number of pups born per litter (**a**,**d**,**g**); number of pups weaned per litter (**b**,**e**,**h**). Survival analysis of pup mortality (**c**,**f**,**i**) during lactation (days postpartum 0.5 to 21.5).

The low dose of fluoxetine did not significantly affect the number of pups born compared to the vehicle; however, the low dose of sertraline and the high dose of both fluoxetine and sertraline caused a reduction in the number of pups born (Figure 2).

In the fluoxetine study, there was a dose-dependent increase in pup mortality during the 21 days postpartum resulting in less pups weaned per litter (Figure 2). The percentage of litters in which all pups died was greater (*p* = 0.0008) for the high dose fluoxetine group (62.5%) compared to the control (20.8%) and low dose (16.0%) groups. The mean number of pups weaned per litter was also reduced by sertraline treatment (low and high doses). However, the number of litters in which all pups died were not different between groups (*p* = 0.6, 5.9 vs. 13.6% for the low dose; *p* = 0.6, 28.6 vs. 72.7% for the high dose).

To further investigate the effect of in utero SSRI exposure on neonatal mortality, we analyzed pup mortality using survival curves. Besides a clear effect of SSRI on neonatal survival, pup mortality occurred primarily before DPP 4.5 independent of treatment (vehicle vs. SSRI) and dose.

### 3.5. Pregnancy Complications

**Figure 3:**
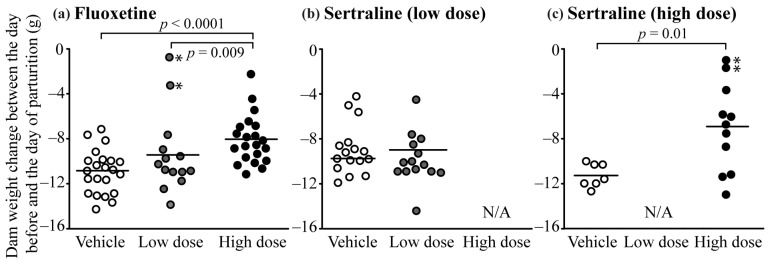
Maternal weight change between the last day of pregnancy and the day of parturition in the fluoxetine (**a**) and sertraline (**b**,**c**) studies. * Denotes dams that were euthanized after parturition and had fully developed dead pups in the uterus.

Some of the SSRI-treated dams that gave birth to an unusually reduced number of pups appeared to still have unborn pups due to visually large abdominal size. A similar finding was not observed in vehicle-treated dams. Four SSRI-treated dams that had all pups die a few days postpartum were euthanized for necropsy on postpartum days 2.5 to 5.5. These dams had 3 to 5 fully developed dead pups still in the uterus. Additionally, one dam from the high dose sertraline group euthanized on postpartum day 21.5 had 3 dead pups in the uterus that appeared to be mummified. Because our experimental design did not anticipate these issues, only a few (4) dams were euthanized and inspected for unborn pups. Consequently, precise quantification of this finding was not possible. However, reporting this finding is important for designing future studies. To gain insight into the incidence of unborn pups in SSRI-treated dams in the present study we examined the maternal weight change between the day before and the day of parturition (Figure 3). Because maternal weight was not different between groups on the day before parturition (except for high dose fluoxetine), a reduced weight loss suggests that fewer pups were born and is indicative of unborn pups. The high dose of both fluoxetine and sertraline had overall less mean weight loss between the last day of pregnancy and the day of parturition. Based on individual maternal weight loss, it seems likely that other SSRI-treated dams with unusually small litter sizes that were not necropsied also had unborn pups after parturition.

## 4. Discussion

Understanding the effects of maternal medication on pregnancy complications and neonatal outcomes is vital to comprehensively assess the risk of perinatal exposure to psychotropic medication on maternal and newborn wellbeing. Herein, we report the effects of two popular antidepressants on pregnancy and neonatal outcomes in a mouse model highlighting a dose-dependent effect of SSRI, particularly fluoxetine, on neonatal outcomes. Interestingly, fluoxetine and sertraline caused comparable reductions in the number of pups born and pup survival despite the distinct placental transfer of each drug; therefore, exposing fetuses to distinct amounts of each drug. This suggests that these adverse neonatal outcomes are likely to be related to the effect of SSRI on the dam and placenta rather than a direct toxic effect of each drug on pup development.

Because SSRI treatments began on DPC 0.5 in the present study, ovulation and fertilization were expected to take place before the onset of treatments and, therefore, to be similar among vehicle and SSRI-treated groups. Additionally, fluoxetine has little to no effect on embryo development in vitro [30] so a direct effect of fluoxetine on embryo development is unlikely. Therefore, the decreased pregnancy per plug in the high dose fluoxetine group (initially observed on DPC 7.5) is likely due to implantation failure. The high dose of fluoxetine could cause implantation failure via multiple mechanisms including: (1) Induction of maternal weight loss that could alter ovarian function [31] disrupting the endocrine environment required for embryo implantation; (2) direct or indirect modulation of estrogen signaling [32,33,34] with consequent disruption of uterine receptivity; or (3) decreased uterine vascular perfusion [10,11,13,18] disrupting uterine vascular remodeling [35]. Previous studies have also indicated an effect of SSRI on embryo implantation and early pregnancy loss in humans [36,37] and animal models [38,39], although the mechanism remains to be elucidated. Since none of the other doses of SSRI had an effect on embryo implantation, the decreased pregnancy rate in the group receiving the high dose of fluoxetine in the present study may be related to a toxic effect of fluoxetine, as discussed later, rather than an expected effect of the drug at therapeutic concentrations in humans. Further studies are needed to confirm this finding and to define the mechanisms and critical period of fluoxetine exposure on impaired embryo implantation to establish the safety of fluoxetine in early pregnancy development.

The reduced maternal weight gain after DPC 7.5 in the high dose of fluoxetine and sertraline-treated groups suggests smaller litter size, reduced embryonic/fetal growth, or both. Indeed, maternal SSRI treatment during gestation has been linked to intrauterine growth restriction in humans [3,6,8,17] and in animal models [38,40]. The mechanisms of SSRI-induced fetal growth restriction have been a prominent area of research worldwide [10]. The SSRI-induced increase in serotonin signaling has been associated with decreased uterine vascular perfusion [13] and vascular lesions on the maternal and fetal sides of the placenta in women [18]. Therefore, maternal exposure to SSRI may compromise placenta function leading to inadequate nutrient exchange between mother and fetus which can result in fetal growth restriction [10]. On the other hand, the number of pups born was reduced in dams exposed to the high dose of fluoxetine and the low and high doses of sertraline suggesting a role for SSRI on embryo implantation and embryonic/fetal survival. However, the unexpected finding of fully developed dead pups still in the uterus days after parturition in SSRI-treated mice further clouds our interpretation of the effects of SSRI on pregnancy establishment and fetal survival. Furthermore, it is not known whether the intrauterine pup death was a cause or a consequence of fetal retention. Although dystocia has not been reported in women taking SSRI during gestation and fluoxetine does not affect uterine contractions [41], it was an unexpected but critical finding in our study and warrants further investigation. In light of this finding, reduced litter size in rodent models treated with SSRI in previous reports and future studies should be interpreted with caution.

Neonatal mortality, primarily during early postnatal period, was increased in all groups exposed to SSRI in the present study. Previous rodent studies have also shown that fluoxetine and sertraline exposure during the perinatal period increase neonatal mortality [23,40,42]. Fetal developmental malformations (primarily cardiac, respiratory, and neurodevelopmental disorders) have been reported as possible causes of neonatal mortality associated with perinatal SSRI exposure [3,23,43,44,45]. However, neonatal mortality may be due to placental insufficiency caused by SSRI disruption of uterine/placental vascular perfusion and structure [3,10,13,18] rather than a direct effect of SSRI on fetal development. Placental insufficiency is generally regarded as the major cause of fetal growth restriction which is associated with perinatal morbidity and mortality. Fetal growth restriction (unrelated to SSRI exposure) leads to several fetal adaptations to restricted nutrient availability including morphological heart changes, increased cardiac workload, and cardiac function issues resembling dilated cardiomyopathy [14]. Interestingly, rodents exposed to SSRI perinatally have altered cardiac morphology [43,46] and dilated cardiomyopathy [23]. Because both drugs promote comparable neonatal mortality and increase serotonin concentrations in the maternal side of the placenta altering placental homeostasis [18,47] but have distinct placental transfer (70% fluoxetine [23] vs. 25% sertraline [24]), placental insufficiency is likely to be the underlying mechanism of SSRI-related pup mortality rather than a direct toxic effect of SSRI on fetal development. Nevertheless, these results do not exclude a direct role of fluoxetine (highest placental transfer) on fetal organogenesis.

The placenta regulates maternal and fetal serotonin homeostasis during gestation [3,48,49,50]. Since embryonic production of sertonin is limited until day 14.5 of gestation in mice, extraembryonic sources of serotonin are required to maintain fetal brain development [48]. SERT located on the apical region of syncytiotrophoblast (maternal side of the placenta) transports maternal-derived serotonin into the placenta during early and mid gestation regulating serotonin content (signaling) on the maternal side of the placenta and providing maternal-derived serotonin needed for fetal development [48,49]. However, during late pregnany serotonin is no longer transported from mother to fetus; instead, organic cation transporter 3 (OCT3) located on the fetal side of the placenta transports fetal serotonin into trophoblasts for degradation [47,49,50]. SSRI inhibition of placental SERT prevents the transport of maternal serotonin into the placenta increasing serotonin signaling on the maternal side of the placenta [47]. Interestingly, OCT3 is inhbited by glucocorticoids [51] and exogenous drugs such as SSRI [47,50]. However, fluoxetine, but not sertraline, decreases the capacity of OCT3 to transport serotonin from fetal circulation into the placenta in an in situ model [47]. Therefore, added to the decreased placental transfer of sertraline [23,24] resulting in lower concentrations of the drug in the fetal circulation, it seems likely that the similar effects of the two drugs on neonatal outcomes are mediated by their common capacity to inhibit SERT on the maternal side of the placenta [47]. This likely leads to increased serotonin signaling with a consequent compromise of placental vascular perfusion and function [11,15].

Systemic concentrations of fluoxetine + norfluoxetine increase after onset of treatment ranging from 160 to 560 ng/mL in humans [21]. In overdosed patients, fluoxetine + norfluoxetine concentrations may reach 1490 ng/mL [52]. In rodent models, although the dose and route of administration of fluoxetine vary among studies, the dose of 20 mg/kg/day via intraperitoneal injection has been widely used [26,27]. Our results clearly demonstrate that this dose produces systemic concentrations that are many fold greater than clinically relevant doses in humans. On the contrary, the low dose of fluoxetine used in our experiment (2 mg/kg/day) resulted in systemic concentrations similar to expected concentrations in humans. Unfortunately, we were unable to measure the sertraline concentration in our study. The overall effects of sertraline (low and high doses) were intermediate compared to the low and high doses of fluoxetine. However, the limitation of sertraline solubility that required increased DMSO concentration and the lack of systemic drug concentrations clouded our full interpretation of the effects of the high dose of sertraline on pregnancy and neonatal outcomes. Although we recognize that extreme dosage treatments are often important for delineating physiologic pathways and investigating possible toxic effects of drugs in animal models, our study highlights the importance of using therapeutic dosages to more accurately evaluate the risk of maternal drug exposure on pregnancy and neonatal outcomes, particularly in translational studies for more direct relevance to human medicine.

Most mice (vehicle and SSRI-treated) in our studies lost weight in the first 24 h after the first treatment. This is expected due to the stress of handling and changing cages for mating, individual housing, and initiation of treatments. However, mice exposed to the high dose of each drug experienced greater weight loss. Nevertheless, mice receiving the high dose of sertraline and low dose of fluoxetine reestablished pretreatment weight by day 2 of treatment (similar to vehicle) while mice receiving the high dose of fluoxetine took 6 days to reestablish pretreatment weight. In animal models, SSRI-induced weight loss has been reported [31,39,40,53]. In humans, short-term fluoxetine treatment is also known to cause weight loss [54]. However, with prolonged treatment, weight gain is most commonly observed. Although the SSRI-induced weight loss in mice may resemble the weight loss observed in short-term fluoxetine treatment in humans, the weight loss in rodents exposed to high doses of SSRI seems to be due to drug overdose because it has been associated with amenorrhea [31], digestive disorders, and death [39,53].

## 5. Conclusions

Overall, our results demonstrate a dose-dependent effect of SSRI exposure during gestation and lactation on pregnancy outcomes and perinatal pup mortality. The comparable neonatal outcomes during treatment with these two drugs that have distinct placental transfer properties make it likely that SSRI-induced placental insufficiency and fetal growth restriction lead to the observed neonatal morbidity/mortality rather than a direct toxic effect of each drug on perinatal mortality. Lastly, we highlight that some effects of treatments with excessive doses of psychotropic medication in animal models (as for the highest dose of fluoxetine in the present study) may not reflect expected effects in humans due to extreme systemic concentrations of the drug, and therefore, should be interpreted with caution.

## Figures and Tables

**Table 1:** Serum concentrations of fluoxetine + norfluoxetine in pregnant mice on DPC 0.75 and 17.75 (6 h after the first and 18th treatments, respectively).

	Vehicle	Low Dose	High Dose	*p* Value
DPC 0.75, ng/mL (range)	0 Undetectable	195.4 ± 14.6 ^b^ (169.5−219.9)	4724.7 ± 354.0 ^a^ (2756.3–5828.4)	<0.0001
DPC 17.75, ng/mL (range)	0 Undetectable	466.8 ± 61.6 ^b^ (336.8–746.6)	20,059.2 ± 2176.5 ^a^ (13,477.8–29,815.6)	<0.0001

^a,b^ indicate significant differences among groups.

**Table 2:** Effect of low and high doses of fluoxetine and sertraline on pregnancy outcomes.

	Vehicle	Low Dose	High Dose	*p* Value
*Fluoxetine*				
Vaginal plug, n	28	32	127	-
Pregnant dams, n	24	25	24	-
Pregnancy per plug, %	85.7 ^a^	78.1 ^a^	18.9 ^b^	<0.0001
Gestation length, day	19.1 ± 0.1	19.1 ± 0.1	18.9 ± 0.1	0.17
*Sertraline low dose*				
Vaginal plug, n	32	32	N/A	-
Pregnant dams, n	22	23	N/A	-
Pregnancy per plug, %	68.8	71.9	N/A	0.99
Gestation length, day	18.8 ± 0.1 ^B^	19.0 ± 0.1 ^A^	N/A	0.096
*Sertraline high dose*				
Vaginal plug, n	11	N/A	16	-
Pregnant dams, n	7	N/A	11	-
Pregnancy per plug, %	63.6	N/A	68.7	0.9
Gestation length, day	18.9 ± 0.3 ^b^	N/A	19.6 ± 0.2 ^a^	0.01

^a,b^ Indicate significant difference among groups. ^A,B^ Indicate significance was approached.

## Data Availability

The data presented in this study are available on request from the corresponding author.
